# The Relationship between Age at Initiation of Regular Drinking of Alcohol and Viral Suppression Status, and Depression Symptoms Among People Living with HIV in South-Western Uganda

**DOI:** 10.1007/s10461-023-04228-4

**Published:** 2023-12-07

**Authors:** Raymond Felix Odokonyero, Robin Fatch, Nneka I. Emenyonu, Debbie M. Cheng, Christine Ngabirano, Julian Adong, Winnie R. Muyindike, Noeline Nakasujja, Carol S. Camlin, Moses Kamya, Judith A. Hahn

**Affiliations:** 1 Department of Psychiatry, School of Medicine, Makerere University College of Health Sciences, Kampala, Uganda; 2 Department of Medicine, University of California, San Francisco, CA USA; 3 School of Public Health, Boston University, Boston, MA USA; 4 Mbarara University of Science and Technology, Mbarara, Uganda; 5 Mbarara Regional Referral Hospital, Mbarara, Uganda; 6 Department of Epidemiology and Biostatistics, University of California, San Francisco, CA USA; 7 Department of Obstetrics, Gynecology & Reproductive Sciences, University of California, San Francisco, CA USA

**Keywords:** Age at First Regular Drinking, Alcohol use, Viral Suppression, Depressive Symptoms, Uganda

## Abstract

Alcohol use is an important factor in achieving and maintaining viral suppression and optimal mental health among persons with HIV (PWH), however, the effect of age at first regular drinking on viral suppression and depression remains poorly understood. Here, using secondary data from the Alcohol Drinkers’ Exposure to Preventive Therapy for Tuberculosis (ADEPT-T) study, we used logistic regression analyses to explore whether there is an association between age at first regular drinking and viral suppression (< 40 copies/ml), or presence of depressive symptoms (Center for Epidemiologic Studies Depression, CES-D ≥ 16) among 262 PWH. The median age at first regular drinking was 20.5 years (IQR: 10), with high proportions starting under age 12 (12.2%) and as teens (13.4%). The majority had an undetectable viral load (91.7%) and 11% had symptoms of probable depression. We found no significant association between age at first regular drinking and viral suppression (i.e., child (aOR = 0.76 95%CI: 0.18, 3.26), adolescent (aOR = 0.74 95%CI: 0.18, 2.97) and young adult (aOR = 1.27 95%CI: 0.40, 3.97)) nor with depressive symptoms (i.e., child (aOR = 0.72 95%CI: 0.19, 2.83), adolescent (aOR = 0.59 95%CI: 0.14, 2.50) and young adult (aOR = 0.57 95%CI: 0.22, 1.53)). Age at first regular drinking among PWH did not appear to be associated with either viral suppression or the presence of depressive symptoms, suggesting interventions may best be focused on the harmful effects of current alcohol use.

## Introduction

Alcohol use is highly prevalent and it affects the HIV continuum of care among persons with HIV (PWH) in sub-Saharan Africa (SSA) [1]. Studies have shown that alcohol use impacts HIV care through delays in testing and starting life-saving antiretroviral therapy (ART) [2], poor adherence to ART, lower levels of viral suppression [3], and negatively impacting HIV transmission and disease progression [4]. Globally, experimental use of alcohol starts in childhood and early adolescence [5, 6]. Although the onset of regular drinking may vary across developmental ages, for many people who use alcohol, first regular drinking starts in childhood (12 years and below) [7] Moreover, early initiation of regular drinking makes young people particularly vulnerable to risky sexual behavior, HIV acquisition, non-adherence to ART, viral non-suppression, and mental problems such as depression [4, 8, 9].

Achieving and maintaining viral suppression among PWH is important because it helps to keep PWH healthy and prevents forward transmission, and it is considered “Treatment as Prevention” (TasP) [10, 11]. However, achievement and maintenance of viral suppression are threatened by continued alcohol use which affects ART adherence [3, 12] [3, 12]. Although alcohol use affects ART adherence which may in turn influence viral suppression, PWH with alcohol use are not more likely to have a detectable viral load [13] It is unclear whether this lack of association may be related to the duration of alcohol use. For example, whether early initiation of regular alcohol use may show different effects on viral suppression rates. No studies exploring this phenomenon were found however, exploring these long-term trends may provide important information. Another important consideration for PWH who drink alcohol is their mental health outcomes such as depression. Depression has been reported in one-third of PWH [14]. Depressive symptoms have been associated with alcohol use [15] and implicated in non-adherence to ART, viral non-suppression, and HIV progression [14], thus it is a key component of HIV morbidity. Although depression is common among PWH and is linked with alcohol use, its association with early initiation of drinking is lacking in published literature.

PWH who start drinking at early ages may have more alcohol use-related complications compared to those that start drinking later [16], which may mean different management and psychosocial support approaches regardless of current alcohol consumption. In HIV programming, to enable an exploration of long-term effects, it may be important to discern between behaviors originating due to young age of initiating regular alcohol use and those which are due to more recent alcohol use. However, no studies have focused on the link between early initiation of regular drinking and viral suppression or depressive symptoms among PWH in rural African settings such as in Uganda.

Uganda has one of the highest levels of the annual consumption of alcohol in Africa. The per capita per year consumption of pure alcohol by people aged 15 years and above in 2016 was 26.0 L [17]. Moreover, a study from the region reported that between 8 and 42% of PWH report engaging in at-risk drinking of alcohol [18]. Uganda also reports a high HIV prevalence of 6.2% among people aged 15–64 years (women − 7.6%; men − 4.7%) [19]. To leverage recently collected lifetime and current alcohol use data in PWH in South-Western Uganda, we explored the relationship between age at first regular drinking and viral suppression and depressive symptoms for those who reported ever drinking alcohol. We conducted secondary data analyses to [1] describe socio-demographic characteristics of age at first regular drinking of alcohol among PWH who have ever consumed alcohol, and [2] determine whether there is an association between age at initiation of alcohol use with viral suppression in this group. We hypothesized that there would be an association between age at first regular drinking and viral suppression status and that early initiation of regular drinking, would reduce the odds of being virally suppressed. We also aimed to [3] determine whether there is an association of age at first regular drinking with the presence of depressive symptoms. We hypothesized that there would be an association between age at first regular drinking and depression symptoms, such that early initiation of regular alcohol use would be associated with higher odds of having depressive symptoms.

## Materials and Methods

This cross-sectional secondary analysis was done using baseline data from a longitudinal cohort study of adult persons with HIV (PWH) attending care at the Mbarara Regional Referral Hospital’s (MRRH) Immune Suppression Syndrome (ISS) clinic in southwestern Uganda. The Alcohol Drinkers’ Exposure to Preventive Therapy for Tuberculosis (ADEPT-T, NCT03302299) study, examined the safety, tolerability, and adherence to isoniazid (INH) preventive therapy (IPT) among PWH who reported current alcohol use (prior 3 months) (n = 200), compared to PWH not engaged in alcohol use in the prior year (n = 100). The ADEPT-T is part of the Uganda-Russia-Boston Alcohol Network for Alcohol Research Collaboration on HIV/AIDS (URBAN ARCH) consortium.

The participants in the ADEPT-T study were recruited and followed from May 2017 – January 2021. Patients were eligible for the study if they were 18 years and above, lived within a 60 km radius or two hours’ drive of Mbarara Regional Referral Hospital, were fluent in either English or Runyankole (the local dialect), showed no evidence of active TB infection according to WHO symptoms criteria, had no prior use of TB medicines for treatment and/or prevention of TB, self-reported current alcohol use (past 3 months) or abstaining (at least 1 year), had alanine aminotransferase (ALT) and aspartate aminotransferase (AST) ≤ 2x of the upper limit of normal (ULN), and were not pregnant (after September 2018) because a study had reported poor birth outcomes among women on IPT in pregnancy [20]. The patient also had to have latent TB detected using skin tuberculin test (> 5 mm within 72 h was considered positive) [21], be living with HIV and enrolled in care from the ISS clinic. For this analysis, only participants who self-reported any lifetime alcohol use were included.

Ethical clearance to conduct the study was obtained from the University of California, San Francisco, Boston University, Mbarara University of Science and Technology, and Mbarara Regional Referral Hospital institutional review boards, and the Uganda National Council of Science and Technology (UNCST). Written informed consent was obtained from all study respondents after explaining the objectives and procedures of the study. Respondents were assured of confidentiality before starting each interview. The secondary analysis was approved by the Makerere University School of Medicine Research and Ethics Committee (Ref: Mak-SOMREC-2021-168).

### Measures

The study questionnaire was translated from English to Runyankole and back-translated. The questionnaire was only used after ascertaining that the original meaning of the questions had not changed during the translation. Data were collected electronically by trained research assistants. The interviews were conducted in the patient’s language of preference (English or Runyankole) in a private room. The questionnaire used at baseline comprised various questions and tools that assessed demographic characteristics, alcohol use, other substance use, medications, physical and mental health status, and several psychosocial measures. The Alcohol Use Disorders Identification Test – Consumption (AUDIT-C) [22], was used for self-reported alcohol use and found positive for scores of ≥ 3 for women and ≥ 4 for men [23]. Alcohol use disorder (AUD) was measured using the diagnostic and statistical manual of mental disorders, 5th edition (DSM-V) [24]. The DSM-V was not administered to those who had abstained from drinking for over 1 year. For this analysis, we considered AUD positive if someone scored 4 or more “yes” responses. The Center for Epidemiologic Studies Depression (CES-D) [25] was used to assess depression. Blood samples were also drawn at baseline for commercial lab testing of PEth, using previously published methods to detect the 160:/18:1 analogue [26], and viral load testing.

### Dependent Variables

The primary outcome in this analysis was viral suppression. We used a cut-off of ≤ 40 copies per milliliter of blood to denote viral suppression “YES” and those > 40 copies as viral suppression “NO”.

The secondary outcome of interest was self-reported depression symptoms over the previous two weeks, measured using the CES-D scale. We were interested in whether depressive symptoms exist or not. A CES-D cut-off of 16 or more warrants clinical attention; as such, we dichotomized ≥ 16 to be “YES” for presence of depressive symptoms and < 16 as “NO” for presence of depressive symptoms [25].

### Independent Variables

Our main independent variable was age at first regular drinking. The age at first regular drinking was obtained from the question: “How old were you when you began to drink at least one drink per month (regular drinking)?” We analyzed age at first regular drinking categorized in the developmental stages comprising “childhood” (< 12 years), “adolescence” (13–17 years), “young adulthood” (18–24 years), and “adulthood” (> 24 years).

### Covariates

The demographic characteristics collected at baseline and examined as potential confounders included: age, sex, education, and marital status. The alcohol use variable examined for potential confounding was current unhealthy drinking, defined as AUDIT-C positive, with a cut-off score of ≥ 3 for women and ≥ 4 for men [23], and/or PEth ≥ 50ng/mL [27].

### Statistical Analysis

Using baseline data for those that reported ever drinking alcohol, we described the demographic characteristics of the sample using frequency distributions for the categorical variables and used the mean, median, and interquartile range for the continuous variables. We described age at first regular drinking overall, as well as by each covariate of interest. Preliminary analyses suggested a non-linear relationship between age at first drinking and the odds of viral suppression and presence of depressive symptoms. Therefore, we conducted subsequent analyses categorizing age at first regular drinking as: Child; Adolescent; Young Adult; Adult.

We used unadjusted and adjusted logistic regression models to examine the associations between age at first regular drinking and (1) viral suppression (primary outcome), and (2) depressive symptoms (secondary outcome). The multivariable models were adjusted for current age, sex, education, marital status, and current unhealthy drinking. In exploratory analyses, we conducted two analyses to examine interaction by gender on the relationship between age at first regular drinking and the two outcomes of interest. We used a significance level of 0.10 to identify interactions.

## Results

A total of 200 persons reporting current alcohol use and 102 persons reporting abstaining for at least 1 year were enrolled in the ADEPT-T study. For this analysis, we included ADEPT-T baseline data from 262 participants with any lifetime use of alcohol reported at baseline. Of these, 141 (53.8%) were male. The median age was 40 years (IQR: 13), about one-quarter (26.3%) had more than primary education, and about two-thirds (67.2%) were married. Out of the 253 participants for whom viral load data were available, 91.7% had an undetectable viral load. The median age at first regular drinking was 20.5 years (IQR: 10). 12% of the sample initiated regular drinking as children (below the age of 12), 13.4% initiated regular drinking as teens (13 to 17 years), 36.6% initiated regular drinking as young adults (18 to 24 years), and 37.8% started regular drinking as adults (25 years and above). More than half, 56.9%, were engaged in current unhealthy drinking. 19% had a diagnosable alcohol use disorder using the DSM-V and 11% reported depressive symptoms (CESD score ≥ 16) (Table 1).

**Table 1:** Participant characteristics of persons with HIV in South-Western Uganda (N = 262)

Participant characteristics	N (%)	Median (Q1, Q3)
Age at baseline		40(34–47)
Sex		
Female	121 (46.2)	
Male	141 (53.8)	
More than a primary education		
No	193 (73.7)	
Yes	69 (26.3)	
Married		
No	86 (32.8)	
Yes	176 (67.2)	
Symptoms of depression		
No	234 (89.3)	
Yes	28 (10.7)	
Undetectable viral load (n = 253)		
No	21 (8.3)	
Yes	232 (91.7)	
Age at first regular drinking		20.5 (17–27)
Child (≤ 12)	32 (12.2)	
Adolescent (13–17)	35 (13.4)	
Young adult (18–24)	96 (36.6)	
Adult (≥ 25)	99 (37.8)	
Current unhealthy drinker*		
No	113 (43.1)	
Yes	149 (56.9)	
Alcohol use disorder**		
No	212 (80.9)	
Yes	50 (19.1)	

*PEth > = 50 and/or AUDIT-C hazardous at baseline

**DSM-5: 4 or more “yes”

The mean (SD) age of initiation of regular drinking overall was 22.1 years (8.1). Participants with detectable viral load had their first regular drinking at a mean age of 20.1 (6.7) while those with undetectable viral load had a mean age of 22.3 (8.2). The mean age for participants with positive depressive symptoms was 21.4 (6.8) and 22.1 (8.3) for those who were negative for depressive symptoms. Participants who had current unhealthy drinking initiated regular drinking at a mean age of 21.8 (8.0) and those without had a mean age of 22.4 (8.3). The mean age of first regular drinking was 20.4 (6.6) among those with AUD and 22.5 (8.4) among those without AUD (Table 2).

**Table 2:** Mean age at first regular drinking by participant characteristics (n = 262)^×^

Participant characteristics	Age at First regular drinking Mean (SD)
Age at baseline	
< 30	18.8 (4.0)
30–39	21.5 (6.0)
40–49	22.7 (8.9)
>=50	24.3 (11.2)
Sex	
Female	22.8 (8.9)
Male	21.5 (7.4)
More than a primary education	
No	21.8 (8.2)
Yes	22.8 (8.1)
Married	
No	22.9 (9.1)
Yes	21.7 (7.6)
Symptoms of depression	
No	22.1 (8.3)
Yes	21.4 (6.8)
Undetectable viral load	
No	20.1 (6.7)
Yes	22.3 (8.2)
Current unhealthy drinker*	
No	22.4 (8.3)
Yes	21.8 (8.0)
Alcohol use disorder**	
No	22.5 (8.4)
Yes	20.4 (6.6)

^×^ ADEPTT study participants at baseline who report ever drinking

*PEth > = 50 and/or AUDIT-C hazardous at baseline

**DSM-5: 4 or more “yes”

In the unadjusted analysis, age at first regular drinking was not significantly associated with viral suppression, nor were other covariates (Table 3). For the primary adjusted analysis, the association between ages at first regular drinking i.e., child (aOR = 0.76 95%CI: 0.18, 3.26), adolescent (aOR = 0.74 95%CI: 0.18, 2.97) and young adult (aOR = 1.27 95%CI: 0.40, 3.97) and viral suppression were not significant (p = 0.84), after adjusting for current age, sex, education, marital status, and current unhealthy drinking. The odds of viral suppression appeared to be greater among participants who had more than primary level education compared to those who did not (aOR = 4.45 95%CI: 0.98, 20.16, p = 0.05).

**Table 3:** Unadjusted and adjusted odds ratios (OR) and 95% confidence intervals (CI) for viral suppression (n = 253)

	Unadjusted OR (95% CI)	*p*-value	Adjusted OR (95% CI)	*p*-value
Age at first regular drinking		0.81		0.84
Child (≤ 12)	0.72 (0.18, 3.00)		0.76 (0.18, 3.26)	
Adolescent (13–17)	0.58 (0.16, 3.14)		0.74 (0.18, 2.97)	
Young adult (18–24)	1.02 (0.34, 3.04)		1.27 (0.40, 3.97)	
Adult (≥ 25)	1.00		1.00	
Age at baseline (per 1 year)	1.02 (0.97, 1.07)	0.42	1.03 (0.98, 1.09)	0.26
Sex		0.23		0.24
Female	1.00		1.00	
Male	0.56 (0.22, 1.45)		0.52 (0.18, 1.53)	
More than a primary education		0.10		0.05
No	1.00		1.00	
Yes	3.54 (0.80, 15.64)		4.45 (0.98, 20.16)	
Married		0.17		0.26
No	1.00		1.00	
Yes	0.46 (0.15, 1.40)		0.51 (0.16, 1.65)	
Current unhealthy drinker		0.71		0.88
No	1.00		1.00	
Yes	0.84 (0.34, 2.11)		1.08 (0.40, 2.95)	

In the unadjusted analysis, age at first regular drinking was not significantly associated with depressive symptoms (Table 4). Marital status was associated with presence of depressive symptoms; the odds of depressive symptoms were significantly lower for those who were married compared to those who were not (aOR = 0.34 95%CI: 0.14, 0.81).

**Table 4:** Unadjusted and adjusted odds ratios (OR) and 95% confidence intervals (CI) for symptoms of depression (n = 262)

	Unadjusted OR (95% CI)	*p*-value	Adjusted OR (95% CI)	*p*-value
Age at first regular drinking		0.93		0.71
Child (≤ 12)	0.75 (0.20, 2.84)		0.72 (0.19, 2.83)	
Adolescent (13–17)	0.68 (0.18, 2.57)		0.59 (0.14, 2.50)	
Young adult (18–24)	0.84 (0.18, 2.05)		0.57 (0.22, 1.53)	
Adult (≥ 25)	1.00		1.00	
Age at baseline (per 1 year)	0.98 (0.94, 1.03)	0.45	0.97 (0.92, 1.02)	0.18
Sex		0.44		0.02
Female	1.00		1.00	
Male	1.37 (0.62, 3.05)		3.27 (1.23, 8.68)	
More than a primary education		0.14		0.09
No	1.00		1.00	
Yes	0.43 (0.14, 1.30)		0.37 (0.12, 1.16)	
Married		0.05		0.02
No	1.00		1.00	
Yes	0.44 (0.20, 0.98)		0.34 (0.14, 0.81)	
Current unhealthy drinking		0.24		0.07
No	1.00		1.00	
Yes	0.62 (0.28, 1.37)		0.44 (0.18, 1.08)	

In the multivariable analysis, the odds of depressive symptoms were not significantly different by age of first regular alcohol use drinking (i.e., child (aOR = 0.72 95%CI: 0.19, 2.83), adolescent (aOR = 0.59 95%CI: 0.14, 2.50) and young adult (aOR = 0.57 95%CI: 0.22, 1.53)) (Table 4). However, there were significant associations between sex and marital status and depressive symptoms. The odds of depressive symptoms were greater for male participants compared to females (aOR = 3.27, 95%CI: 1.23, 8.68, p = 0.02). In addition, the odds of depressive symptoms were lower for participants who were married compared to those who were not (aOR = 0.34, 95%CI: 0.14, 0.81, p = 0.02).

## Discussion

While there was a high percentage of persons reporting early (childhood and early adolescent) alcohol use, the unadjusted and adjusted analyses from our study did not support the primary hypothesis that there is an association between age at first regular drinking and viral suppression, nor did we find that the association differed by gender. These findings were counter to our hypotheses, but we did not find any prior studies in the literature for comparison. The available literature focused on current alcohol use, such as in Foley et al., [28] and Cerutti et al. [4], or on alcohol use disorder [4], as correlates of viral non-suppression. Our exploration of the association between age at first regular drinking, while focusing on the developmental age at which it was started, adds a new aspect to the literature. A possible reason for finding no association in our analysis may be because age at first regular drinking of alcohol may have its effect on viral suppression much earlier in the continuum of care but our study participants had already been on ART for at least 6 months. Their engagement in care, which includes adherence counselling, may have limited the effects of alcohol use in ART adherence. In addition, our sample was mostly comprised of middle-aged people (median age of 40 years), therefore it is possible that the effect of age at first regular drinking on viral suppression had diminished. This is consistent with Labouvie and White’s proposal that age of onset of alcohol use may be a useful indicator of risk of abuse and dependence when the period of observation is limited to adolescence or young adulthood, but that over time other factors supersede the age of onset in importance [29].

We were also unable to support our secondary hypothesis that there is an association between age at first regular drinking and depressive symptoms, and the association did not appear to differ by gender. The proportion with depression in our sample of PWH who reported ever drinking alcohol is 11%, lower than the estimate by Foley et al., in the same setting in which they reported the prevalence of depressive symptoms among PWH with hazardous alcohol use to be 27% [28]. Depression symptoms could be lower because only 57% of our sample was currently an “unhealthy” drinker. Some studies have reported an association between alcohol use and psychosocial outcomes such as depression [30, 31] but not all [28]. Although we focused on the link between age at first regular drinking of alcohol and depressive symptoms, and therefore cannot make a clear comparison with studies focused on current alcohol use patterns, our data equally showed no association between current alcohol use with viral suppression or depression. It is possible that among PWH in care, alcohol use generally decreases as patients move through the continuum of care such that various interventions, whether intentional or not, mitigate the effects of drinking history and current alcohol use on viral suppression and depression. Whereas our study reports no difference in associations between age at first regular drinking and depressive symptoms by gender, we found gender differences in depression symptoms on their own, with males more likely to have depressive symptoms than females. This is a surprising result given that depressive symptoms have been more commonly reported among women than men. A possible reason may be the fact that women in this setting engage better with HIV care services and as such are more likely to talk about their distress, and get assessed and managed for depressive symptoms compared to men. This calls for interventions that target men with HIV for the identification and management of depressive symptoms.

There were some limitations in our study to report. Firstly, this being a secondary data analysis, the data was not intended to assess the associations in our hypothesis. Consequently, the numbers of participants reporting early alcohol use, unsuppressed viral load, and depressive symptoms were relatively small, limiting statistical power. However, the estimates obtained by this research will be useful for the design of future studies. Future research should look to screen for both depression and age at first drinking much earlier in the HIV care continuum in order to get an accurate assessment of the associations between age at first regular drinking, viral suppression and depression. Secondly, our age at first regular use variable was self-reported; age at first use appears to increase with current age (Table 2), suggesting possible recall and social desirability bias in self-report. Thirdly, we categorized our variables for easy interpretability but may have introduced some bias in the process.

This study has the following strengths. First, we are unaware of similar studies in our setting that assessed the relationship between age at first regular drinking and HIV care outcomes such as viral suppression and depressive symptoms. Second, although not applicable to this analysis, social desirability and recall bias were mitigated in the assessment of current unhealthy alcohol use using self-reports and an alcohol biomarker (PEth) in the rest of the study.

In conclusion, while many persons in our sample started regular alcohol consumption at an early age, age at first regular drinking among PWH did not appear to be associated with either viral suppression status or depressive symptoms but the point estimates appear clinically notable despite the wide intervals, therefore interventions can focus on the harmful effects of current alcohol use, keeping in mind the above limitations.

We recommend that future studies that prospectively focus on age at initiation of regular drinking at earlier stages of the HIV continuum of care i.e., prevention, testing and ART initiation. This will enable examination of the temporal relationship between age at initiation and viral suppression and depression. We also recommend qualitative exploration of the relationship between age at initiation of regular drinking and viral suppression and depression in this population.

## Data Availability

Data analyzed for this manuscript will be made available via the Dryad Digital Repository upon acceptance for publication.
